# Identification of Metabolism-Related Proteins as Biomarkers of Insulin Resistance and Potential Mechanisms of m^6^A Modification

**DOI:** 10.3390/nu15081839

**Published:** 2023-04-11

**Authors:** Yan-Ling Li, Long Li, Yu-Hong Liu, Li-Kun Hu, Yu-Xiang Yan

**Affiliations:** 1 Department of Epidemiology and Biostatistics, School of Public Health, Capital Medical University, Beijing 100069, China; 2 Beijing Municipal Key Laboratory of Clinical Epidemiology, Beijing 100069, China; 3 Department of Neurosurgery, Xuanwu Hospital, Capital Medical University, Beijing 100053, China; 4 China International Neuroscience Institute (China-INI), Beijing 100053, China

**Keywords:** type 2 diabetes, insulin resistance, m^6^A modification, bioinformatics analysis

## Abstract

Background: Insulin resistance (IR) is a major contributing factor to the pathogenesis of metabolic syndrome and type 2 diabetes mellitus (T2D). Adipocyte metabolism is known to play a crucial role in IR. Therefore, the aims of this study were to identify metabolism-related proteins that could be used as potential biomarkers of IR and to investigate the role of N^6^-methyladenosine (m^6^A) modification in the pathogenesis of this condition. Methods: RNA-seq data on human adipose tissue were retrieved from the Gene Expression Omnibus database. The differentially expressed genes of metabolism-related proteins (MP-DEGs) were screened using protein annotation databases. Biological function and pathway annotations of the MP-DEGs were performed through Gene Ontology and Kyoto Encyclopedia of Genes and Genomes pathway analyses. Key MP-DEGs were screened, and a protein–protein interaction (PPI) network was constructed using STRING, Cytoscape, MCODE, and CytoHubba. LASSO regression analysis was used to select primary hub genes, and their clinical performance was assessed using receiver operating characteristic (ROC) curves. The expression of key MP-DEGs and their relationship with m^6^A modification were further verified in adipose tissue samples collected from healthy individuals and patients with IR. Results: In total, 69 MP-DEGs were screened and annotated to be enriched in pathways related to hormone metabolism, low-density lipoprotein particle and carboxylic acid transmembrane transporter activity, insulin signaling, and AMPK signaling. The MP-DEG PPI network comprised 69 nodes and 72 edges, from which 10 hub genes (*FASN*, *GCK*, *FGR*, *FBP1*, *GYS2*, *PNPLA3*, *MOGAT1*, *SLC27A2*, *PNPLA3*, and *ELOVL6*) were identified. *FASN* was chosen as the key gene because it had the highest maximal clique centrality (MCC) score. *GCK*, *FBP1*, and *FGR* were selected as primary genes by LASSO analysis. According to the ROC curves, *GCK*, *FBP1*, *FGR*, and *FASN* could be used as potential biomarkers to detect IR with good sensitivity and accuracy (AUC = 0.80, 95% CI: 0.67–0.94; AUC = 0.86, 95% CI: 0.74–0.94; AUC = 0.83, 95% CI: 0.64–0.92; AUC = 0.78, 95% CI: 0.64–0.92). The expression of *FASN*, *GCK*, *FBP1*, and *FGR* was significantly correlated with that of *IGF2BP3*, *FTO*, *EIF3A*, *WTAP*, *METTL16*, and *LRPPRC* (*p* < 0.05). In validation clinical samples, the *FASN* was moderately effective for detecting IR (AUC = 0.78, 95% CI: 0.69–0.80), and its expression was positively correlated with the methylation levels of *FASN* (r = 0.359, *p* = 0.001). Conclusion: Metabolism-related proteins play critical roles in IR. Moreover, *FASN* and *GCK* are potential biomarkers of IR and may be involved in the development of T2D via their m^6^A modification. These findings offer reliable biomarkers for the early detection of T2D and promising therapeutic targets.

## 1. Introduction

Insulin resistance (IR) is a significant pathophysiological basis and necessary stage of type 2 diabetes (T2D) as well as a critical pathogenic component of the metabolic syndrome [1]. The condition may develop as early as 13 years prior to the clinical diagnosis of T2D [2]. Therefore, the precise identification of IR and the provision of appropriate interventions for high-risk groups could effectively delay or prevent its development.

The pathogenesis of IR involves the attenuation or blocking of insulin signal transition as well as impaired glycolipid, lipid, and energy metabolism [2,3]. Under normal body conditions, elevated glucose levels in the body stimulate insulin secretion by islet β-cells. The insulin molecules then circulate to the target tissue via the blood system and bind to the insulin receptors on the tissue cell membrane. Next, a series of specific signals trigger the cells to produce biological effects that promote glucose intake and use, thereby reducing the blood glucose level. Importantly, the phosphoinositide 3-kinase/protein kinase B (PI3K/Akt) signaling pathway is a key pathway for insulin signal transduction. T2D occurs when there is insufficient insulin secretion to compensate for IR, thereby disrupting signal transduction in pathways involved in the uptake and usage of insulin by target tissues [2,4]. The effector organs of IR are the liver, skeletal muscles, and adipose tissues. In the liver, IR is characterized by increased glucose production and impaired glucose uptake [5]. By contrast, reduced glucose uptake is the defining feature of IR in adipose tissue and skeletal muscles [4,6], the latter of which are the primary sites of glucose disposal. Additionally, IR occurs in adipocytes, whereas muscle IR-KO mice are typically healthy.

Adipocytes play a crucial role in the regulation of mammalian metabolic homeostasis. These cells have a significant impact on energy expenditure, glucose and lipid balance, and IR development [5,6]. Adipocyte dysfunction causes the dysregulation of adipokines, which may influence inflammatory responses, glucose and lipid homeostasis, and other metabolic illnesses, including diabetes [6,7,8]. Adiponectin and leptin, which are mainly expressed in adipose tissue, can promote energy consumption and metabolism, reduce fat accumulation, and improve insulin sensitivity [9,10]. Although fibroblast growth factor 21 (FGF21) is known to regulate glucose metabolism [11], the molecular mechanisms underlying IR are not fully understood. Identifying biomarkers for the early and accurate diagnosis and prevention of T2D and conducting further research focusing on the molecular mechanism of IR are necessary.

Bioinformatics analysis of transcriptional profiles using RNA sequencing (RNA-seq) methods is an innovative technique for investigating disease pathophysiology, identifying disease biomarkers, and proposing therapeutic targets [12,13]. According to several transcriptomic profiling studies, the gene expression profiles in peripheral blood cells of patients with T2D vary considerably, indicating that the proteins encoded by differentially expressed mRNAs might play a role in the etiology of the disease [14,15]. The integration of bioinformatics methodologies and expression profiling techniques may help in determining novel biomarkers and discovering the molecular pathogenesis of T2D.

Recently, the N^6^-methyladenosine (m^6^A) modification of mRNA has been proposed to play a vital role in the progression of T2D [16,17,18]. The aims of this study were to identify metabolism-related proteins that could function as potential biomarkers of IR and to elucidate the role of m^6^A modification in the pathogenesis of this condition. The results of this study may contribute reliable biomarkers for the early detection and prevention of T2D.

## 2. Materials and Methods

### 2.1. Design of the Study

**Figure 1:**
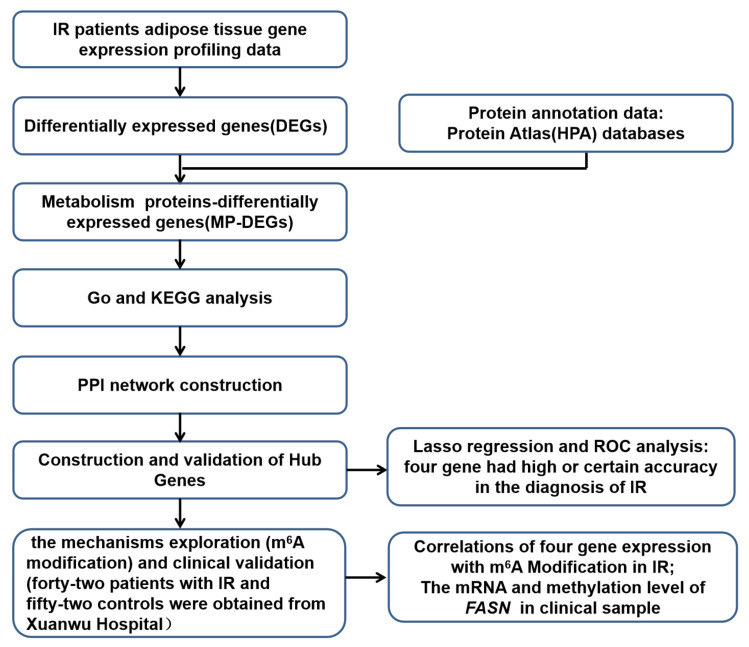
Flowchart of the study design.

First, the RNA-seq dataset GSE174475 from the Gene Expression Omnibus (GEO) database was used to identify differentially expressed genes (DEGs) between patients with IR and matched healthy individuals (controls). Second, DEGs coding for metabolism-related proteins (MP-DEGs) were selected. Functional and pathway enrichment analyses of the MP-DEGs were then carried out using the Gene Ontology (GO) and Kyoto Encyclopedia of Genes and Genomes (KEGG) databases. Third, a protein–protein interaction (PPI) network of the MP-DEGs was constructed and used to select functional modules and hub genes. Subsequently, 10 hub genes were selected for further analysis. Key hub genes were further identified using the least absolute shrinkage and selection operator (LASSO) regression analysis method, and their clinical value for the diagnosis of IR was evaluated using receiver operating characteristic (ROC) curves. Furthermore, the relationships between candidate gene expression and m^6^A modification were explored. Finally, adipose tissues from patients with IR and healthy individuals were collected to validate their expression of the key MP-DEGs and their correlation with m^6^A modification. A flowchart of this study is shown in Figure 1.

### 2.2. Data Acquisition and Processing

The RNA-seq dataset GSE174475, which contains data on pre-processed adipose tissue samples from 27 patients with IR and 16 healthy individuals (controls), was downloaded from the GEO database [19]. The BioBase package was used to normalize the data.

### 2.3. Selection of MP-DEGs

The limma package in R is a powerful tool for assessing DEGs [20]. We used this tool to examine the DEGs between the patient and control tissues in the GSE174475 dataset (detection criteria: *p* < 0.05 and |log2 fold change| ≥ 1).

The list of genes coding for metabolism-related proteins was downloaded from the Human Protein Atlas (HPA) protein annotation database [21]. The DEGs screened using limma were then intersected with the genes on the HPA list to screen out the MP-DEGs, and these were then examined for differences in expression between the two tissue groups.

### 2.4. Functional and Pathway Enrichment Analyses of the MP-DEGs

The GO and KEGG enrichment analyses of the MP-DEGs were conducted using the clusterProfiler package in Bioconductor. We first identified the GO biological processes (BPs), cellular components (CCs), and molecular functions (MFs) of the MP-DEGs using the human genome as a background reference (cutoff values: *p* < 0.05 and count = 2). After classifying the MP-DEGs into upregulated and downregulated groups, KEGG pathway enrichment analysis was conducted for each group, using a *p-*value of less than 0.05 and a count of 2 or higher as cutoff values. Finally, the findings of the enrichment study were displayed as dot plots and bar plots using the clusterProfiler package.

### 2.5. Construction of the Protein–Protein Interaction Network of the MP-DEGs and Gene Expression Analysis

PPIs in functional protein association networks were evaluated with the STRING database, using core factors as question proteins. A PPI network of the MP-DEGs was built on the basis of STRING11.5 [22]. Cytoscape was used to visualize the network, with a confidence score of greater than 0.4, and the disconnected genes were concealed. Text mining data were used to filter out interactions that have been confirmed in published literature. To identify functional clusters of genes in the PPI network, Molecular Complex Detection (MCODE) in Cytoscape was used, with a degree cutoff of 2, node score cutoff of 0.2, k-core of 2, and maximum depth of 100 [23]. Modules with proven scores greater than 5 were eliminated. The top 10 node genes were identified using 10 different analytical methods in the CytoHubba plug-in in Cytoscape, and the hub genes were then screened using the intersection of the results. In CytoHubba, the maximal clique centrality (MCC) technique is the most accurate one [24].

### 2.6. LASSO Regression Analysis

LASSO analysis was performed using the glmnet package in R, and the most advantageous cost of the penalty parameter was determined via 10-fold cross-validation [25]. First, the glmnet package was used to create a model with “binomial” as the family setting. Next, the important variables were selected according to the coefficients in the abovementioned model. Then, “deviance” was used as a minimum target parameter in the cross-verification fitting model. Finally, the target variables were selected on the basis of the variable coefficient and the optimal variables of the optimal lambda.

### 2.7. Clinical Sample Collection and Processing

Human cervical adipose tissues from 42 patients with IR and 52 controls were obtained from Xuanwu Hospital, Capital Medical University (Beijing, China) from December 2021 to December 2022. Peripheral venous blood samples of 10 mL were collected. Peripheral venous blood samples of 5 mL were used to measure fasting plasma glucose (FPG), triglycerides (TG), total cholesterol (TC), high-density lipoprotein cholesterol (HDL-C), and low-density lipoprotein cholesterol (LDL-C) by an automated chemistry analyzer (7600, Tokyo, Hitachi, Japan), and plasma FIns levels by aγ-automatic radioimmunoassay counter (XH-6020, Hitachi, Japan). All tissue samples and clinical data were collected with each patient’s consent. The study protocol was approved by the Ethics Committee of Capital Medical University (Approval No. 2017SY24).

All adipose tissue samples were stored in a refrigerator at −80 °C. Total RNA was extracted from each sample using the SV Total RNA Isolation System (Z3100, Promega, Madison, WI, USA) according to the manufacturer’s instructions. The reverse transcription-quantitative polymerase chain reaction (RT-qPCR) was performed on a QuantStudio5 system (Applied Biosystems, Thermo Fisher, Waltham, MA, USA) using the RT2 SYBR Green ROX FAST Mastermix (Qiagen, Germantown, MD, USA). The gene-specific primers used are shown in Supplementary Table S1. GAPDH was used as an endogenous reference to normalize the relative amount of each target gene. The expression level was calculated using the 2^−∆∆CT^ method.

### 2.8. Grouping of Study Subjects

The study subjects of this experiment were divided into an insulin-resistant-positive group and a normal control group. The insulin resistance index was evaluated by the homeostasis model assessment (HOMA) and the HOMA-IR (homeostasis model assessment insulin resistance) index. HOMA-IR value >2.5 is IR positive [26]. *HOMA-IR =* $\frac{FIns\times PG}{22.5}$. The IR group was IR positive, and individuals were excluded from the study based on exclusion criteria. The exclusion criteria were as follows: patients with T2D; patients with coronary heart disease and myocardial disease; patients with malignant tumors; patients with acute and chronic infectious diseases; patients with severe liver and kidney diseases; those who use drugs to lower glucose; and those on hormones, psychotropics, and other prescription medications. The normal control group was IR negative, and individuals were excluded from the study based on exclusion criteria. HOMA-IR value <2.5 is IR negative. The exclusion criteria were as follows: patients with T2D; patients with coronary heart disease and myocardial disease; patients with malignant tumors; patients with acute and chronic infectious diseases; patients with severe liver and kidney diseases; and those who use drugs to lower glucose; those on hormones, psychotropics, and other prescription medications.

### 2.9. Gene-Specific MeRIP-qPCR

The EpiQuik^TM^ CUT&RUN m^6^A RNA Enrichment Kit (A-P-9018, EpiGentek, Farmingdale, NY, USA) was used to perform the m^6^A-based methylated RNA immunoprecipitation (MeRIP) assay according to the manufacturer’s instructions. Next, using an mRNA purification kit (Z5300; Promega, USA), a portion of the total RNA was converted into mRNA. The enriched RNA was immediately quantified using qPCR after enrichment of the methylated RNA with an m^6^A antibody. In brief, A/G immunomagnetic beads were first mixed with m^6^A antibodies, following which the mixture was added to the mRNAs. Next, the m^6^A immunomagnetic beads were enriched using a magnetic frame, and the enriched RNA–antibody complexes were digested with protease. Finally, routine qPCR was performed for quantification of the mRNAs. The primer sequences used for the qPCR are listed in Supplementary Table S1.

### 2.10. Statistical Analysis

DEGs were examined using the limma package in R (version 4.0). The PPI network and hub genes were analyzed using Cytoscape 8.0. GO and KEGG enrichment analyses of the MP-DEGs were conducted using the clusterProfiler package in Bioconductor. LASSO regression analysis was performed using the glmnet package in R. Correlations between *FASN*, *GCK*, *FBP1*, and *FGR* and m^6^A-related genes were analyzed using Pearson’s correlation coefficient for normal data and Spearman’s test for non-normal data. The average mRNA and methylation levels are expressed as the mean ± standard deviation. Differences between groups were compared using the *t*-test. Statistical significance was set at a *p*-value of less than 0.05.

## 3. Results

### 3.1. Identification of Differentially Expressed Genes

**Figure 2:**
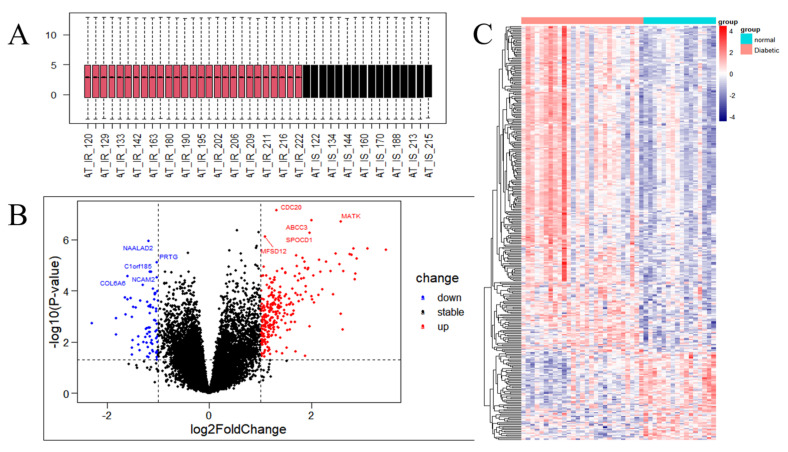
Analysis of the gene expression correlation and differential gene expression between the insulin resistance (IR) and control groups in the dataset. (**A**) Box plot of the gene probe expression levels among the samples. There was no significant difference in the median or upper and lower quartiles. (**B**) Volcano map of all differentially expressed genes (DEGs) in the IR and control groups analyzed with the limma R package; *p* < 0.05 and |log2 fold change| ≥ 1. The top 10 upregulated and downregulated genes with the smallest *p*-values are marked on the map. (**C**) Heatmap of all DEGs in the IR and control groups.

DEG analysis was performed to assess differences between the IR and control groups of the GSE174475 dataset. Basic information of samples for GSE174475 used in the study is presented in Supplementary Table S2. The genes in this dataset had the same quartile, Max, and Min values (Figure 2A). In total, 344 genes were identified as DEGs (Supplementary File S1). *CDC20*, *ABCC3*, *MATK*, *SPOCD1*, and *MFSD12* were the top five upregulated genes, whereas *NAALAD2*, *PRTG*, *Clorf185*, *NCAM2*, and *COL6A6* were the top five downregulated ones, with the lowest *p*-values (Figure 2B). The heatmap showed that the DEGs were consistently significantly upregulated or downregulated in the IR group relative to their expression levels in the control group (Figure 2C).

### 3.2. Selection of the MP-DEGs

**Figure 3:**
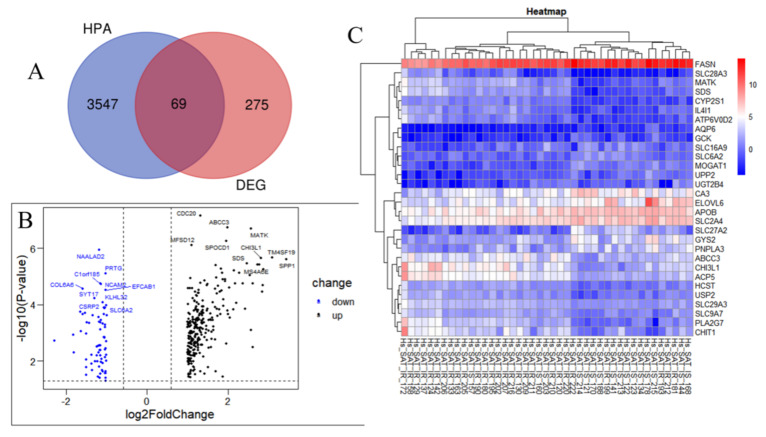
Screening of differentially expressed genes (DEGs) encoding metabolism-related proteins. (**A**) The genes encoding metabolism−related proteins annotated in the Human Protein Atlas (HPA) database were intersected with the 344 DEGs, whereupon 69 differentially expressed genes of metabolism−related proteins (MP−DEGs) were screened out. (**B**) Volcano map of MP−DEGs in the IR and control groups. The top 10 upregulated and downregulated genes with the smallest *p*−values are marked. (**C**) Heatmap of the top 15 upregulated and downregulated MP−DEGs with the smallest *p*−values.

Annotated metabolism-related genes from the HPA database were used to identify the genes encoding metabolism-related proteins among the DEGs between the IR and control groups. After intersecting the HPA-sourced list with the DEGs identified from the GSE174475 dataset, 69 MP-DEGs were filtered out, of which 15 were upregulated and 54 were downregulated (Figure 3A and Supplementary File S2). *ABCC3*, *MATK*, *CHI3L1*, *SDS*, *ACP5*, *IL4I1*, *PLA2G7*, *SLC29A3*, *ATP6V0D2*, and *SLC9A7* were the top 10 upregulated genes, whereas *SLC6A2*, *CA3*, *AQP6*, *APOB*, *SLC2A4*, *GCK*, *SLC27A2*, *MOGAT1*, *SLC16A9*, and *FASN* were the top 10 downregulated ones (Figure 3B and Supplementary File S2). Among the top 15 significantly downregulated genes in IR, *SLC6A2*, *CA3*, *AQP6*, *APOB*, and *SLC2A4* had the smallest *p*-values according to the heatmap of the MP-DEGs (Figure 3C).

### 3.3. Functional and Pathway Enrichment of the MP-DEGs

**Figure 4:**
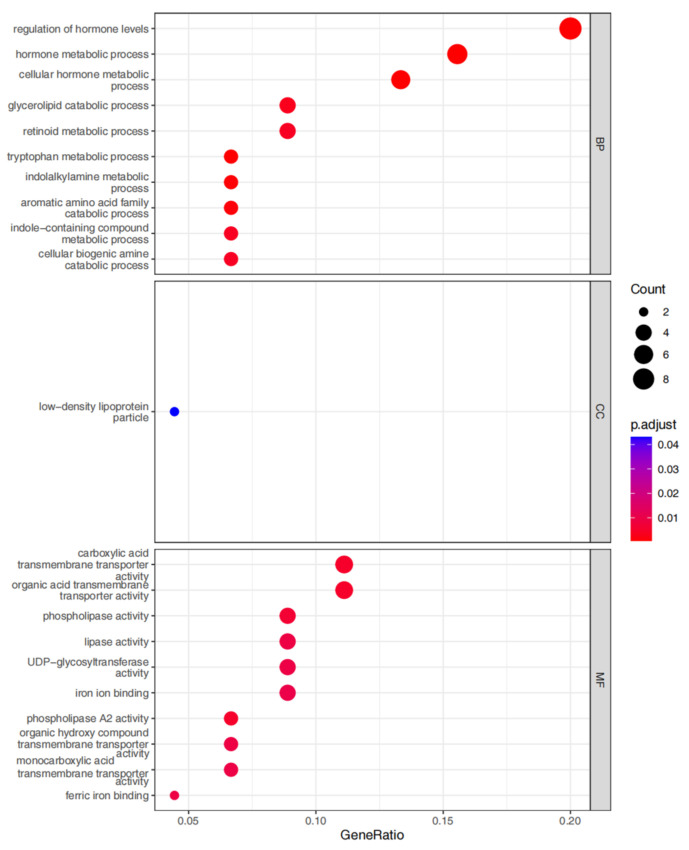
Gene ontology (GO) enrichment analysis of the differentially expressed genes of metabolism-related proteins (MP-DEGs). The dot plots show the top 10 processes enriched in the MP−DEGs in the Biological Process (BP), Cellular Compartment (CC), and Molecular Function (MF) categories.

**Figure 5:**
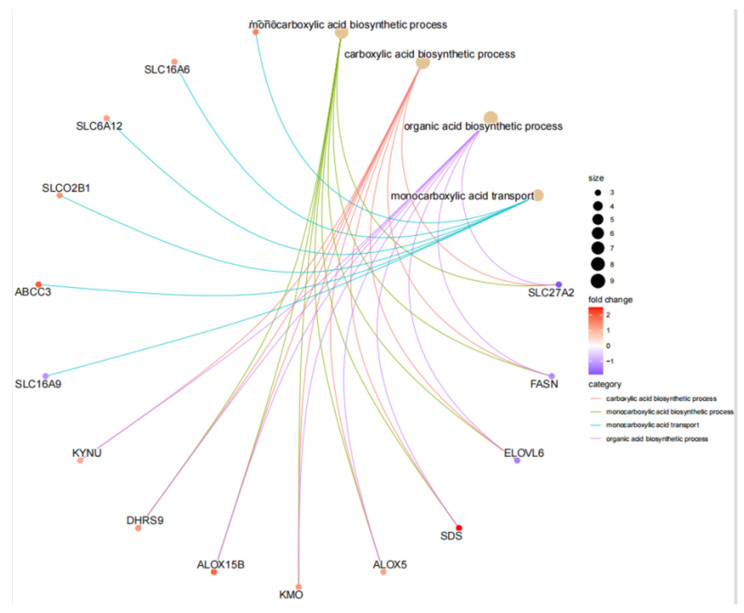
Circle graph from the gene ontology (GO) enrichment analysis of the differentially expressed genes of metabolism-related proteins (MP−DEGs).

**Figure 6:**
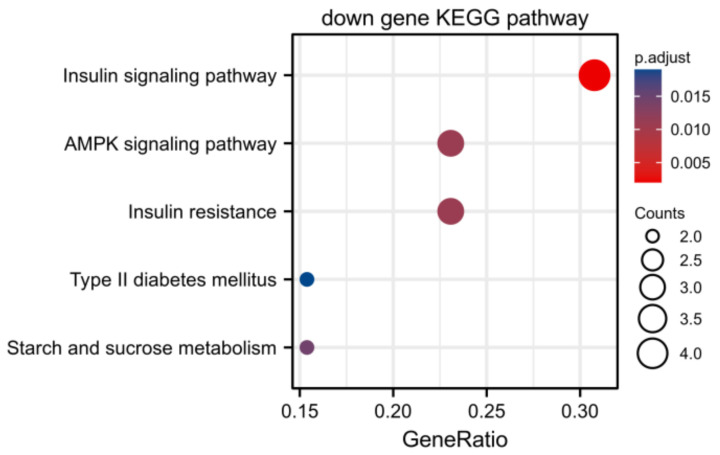
Kyoto Encyclopedia of Genes and Genomes (KEGG) enrichment analysis of the differentially expressed genes of metabolism-related proteins (MP−DEGs) showing the pathways in which the downregulated genes are enriched.

The screened MP-DEGs were mainly enriched in the GO BP categories regulation of hormone levels and hormone metabolic process, the CC category low-density lipoprotein particles, and the MF categories carboxylic acid transmembrane transporter activity and organic acid transmembrane transporter activity (Figure 4). The MP-DEGs were shown using the cnetplot function in the clusterProfiler package. *SLC27A2*, *FASN*, *ELOVL6*, *SLC16A9*, *SLC6A12*, and *SDS* were enriched in multiple terms of the BP category (Figure 5 and Supplementary File S3). KEGG pathway analysis was used to determine the pathways enriched in the upregulated and downregulated genes. The upregulated genes were not enriched in any pathway. By contrast, the downregulated genes were enriched in the insulin signaling, IR, and AMPK signaling pathways (Figure 6).

### 3.4. Protein–Protein Interaction Network Construction and Module Analysis

**Figure 7:**
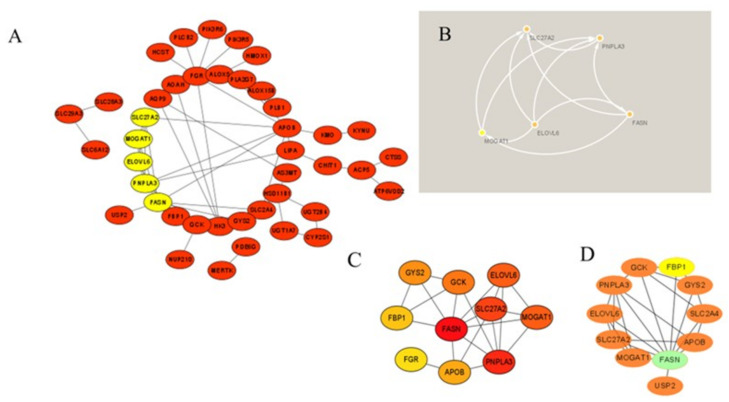
Protein−protein interaction (PPI) network of the differentially expressed genes of metabolism-related proteins (MP−DEGs) and screening of hub genes. (**A**) The STRING database was used to construct the PPI network of MP−DEGs, with 69 nodes and 72 edges. (**B**) The node gene cluster with the highest score, constructed using the MCODE plug-in in Cytoscape, consists of nine genes. (**C**) CytoHubba was used to construct the top 10 hub genes. The figure shows the top 10 hub genes constructed with the maximal clique centrality (MCC) method. (**D**) CytoHubba was used to predict the first−stop node genes that interact with *FASN*. In total, 10 genes were predicted, of which one was upregulated and nine were downregulated.

The STRING database was used to create a PPI network from the 69 MP−DEGs, following which the network was used to explore the interactions between the metabolism-related proteins. Cytoscape was used to visualize the entire PPI network (Figure 7A). The PPI network comprised 72 edges and 69 nodes. The MCODE plug-in in Cytoscape was used to create the functional modules. The findings revealed that only one module, comprising 9 genes and 34 edges, had an established score of greater than 5 (Figure 7B). The top 10 hub genes were screened using the 10 topological approaches of the CytoHubba plug-in in Cytoscape. Ten genes were identified using all ten methods, namely, *FASN*, *GCK*, *FGR*, *FBP1*, *GYS2*, *PNPLA3*, *MOGAT1*, *SLC27A2*, *PNPLA3*, and *ELOVL6* (Figure 7C and Supplementary File S4). *FASN* was selected as the key gene because it had the highest MCC score. The first node gene that interacted with *FASN* was determined using CytoHubba. Of the 10 genes screened, 1 was upregulated and 9 were downregulated (Figure 7D).

### 3.5. Selection and Validation of the Primary Hub Genes

**Figure 8:**
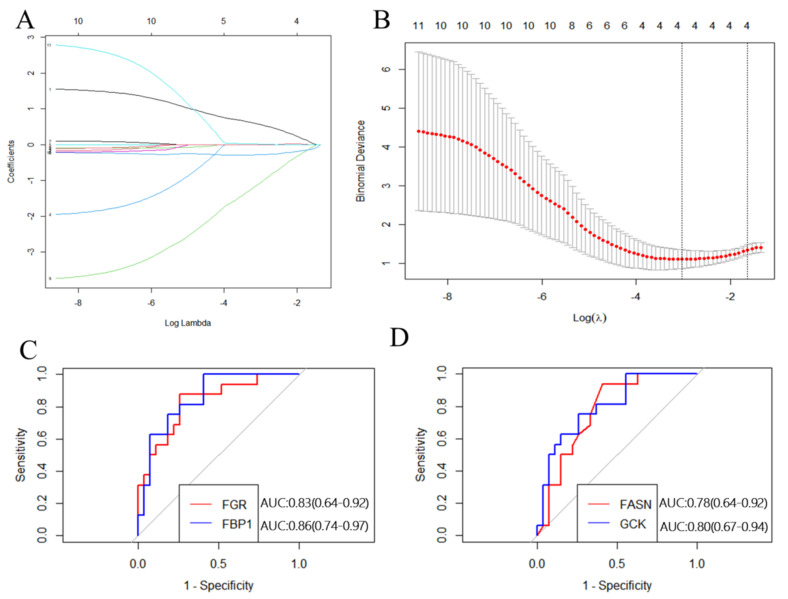
Construction and validation of the hub genes. (**A**) Plots of LASSO regression coefficients versus different values of the penalty parameter. (**B**) Cross−validation plot for the penalty term. (**C**) ROC curves for *FGR* and *FBP1* (AUC: 0.83 and 0.86, respectively); *p* < 0.05. (**D**) ROC curves for *FASN* and *GCK* (AUC: 0.78 and 0.80, respectively); *p <* 0.05.

The 10 hub genes identified from the PPI network were submitted to LASSO regression analysis to identify the primary hub genes, whereupon *GCK*, *FBP1*, and *FGR* were selected in the model (Figure 8). These three genes, as well as *FASN* (another key gene in the PPI network), were studied further using ROC curve analysis. *GCK*, *FBP1*, and *FGR* were highly accurate in diagnosing IR (AUC = 0.80, 95% CI = 0.67–0.94; AUC = 0.86, 95% CI = 0.74–0.94; AUC = 0.83, 95% CI = 0.64–0.92), whereas *FASN* showed moderate accuracy in this regard (AUC = 0.78, 95% CI = 0.64–0.92; Figure 8). These results indicate that *FASN*, *GCK*, *FBP1*, and *FGR* can be applied as reliable biomarkers of IR, with the expression of *FASN*, *GCK,* and *FBP1* being downregulated and that of *FGR* being upregulated in patients with this condition.

### 3.6. FASN Is Lower in IR and Associated with FPG and HbA1c

**Figure 9:**
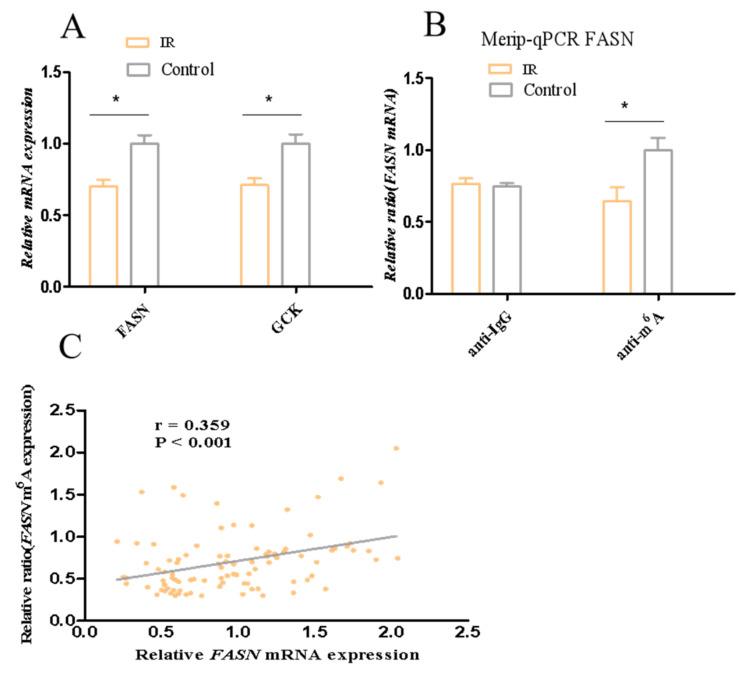
mRNA levels of *FASN* and *GCK* and the methylation level of *FASN*. (**A**) mRNA levels of *FASN* and *GCK*. (**B**) The methylation level of *FASN*. (**C**) Spearman correlation analysis between mRNA levels of FASN and methylation level of *FASN* in all participants. * *p <* 0.05.

To verify the *FASN* and *GCK* diagnostic values for IR, the mRNA levels of *FASN* and *GCK* for adipose tissue were detected in clinical samples from 42 patients with IR and 52 controls. Clinical information of the samples is presented in Supplementary Table S3. The *FASN* and *GCK* mRNA expression was lower in patients with IR than in the controls (Figure 9A, *p <* 0.05). Moreover, *FASN* mRNA expression was negatively correlated with FPG, HbA1c, Homa-IR, and FIns (r = −0.309, *p* = 0.002; r = −0.296, *p* = 0.004; r = −0.279, *p* = 0.006, r = −0.325, *p* = 0.001) in all participants (Table 1). *GCK* mRNA expression was negatively correlated with FPG and Homa-IR (r = −0.220, *p* = 0.017; r = −0.230, *p* = 0.022) in all participants (Table 1). The strength of the association between the mRNA levels of *FASN, GCK,* and IR was evaluated by univariate and multivariate regression models. The univariate logistic model showed that *FASN* and *GCK* were associated with IR. Subsequently, several multivariate logistic regression models were established by adjusting for covariates. The multivariate logistic models showed that *FASN* and *GCK* were also associated with IR, indicating that the association of *FASN* and *GCK* with IR was independent (Supplementary Table S4). We further evaluated the ability of the *FASN* and *GCK* to predict IR as a biomarker by ROC analysis. The results showed that the *FASN* was moderately effective for the detection of IR, with the area under the curve being 0.78 and the cutoff value being 0.33% (Table 2 and Supplementary Figure S1). These results indicated that *FASN* could be used as a reliable biomarker of IR.

### 3.7. Correlations of Candidate Gene Expression with m^6^A Modification in Insulin Resistance

**Figure 10:**
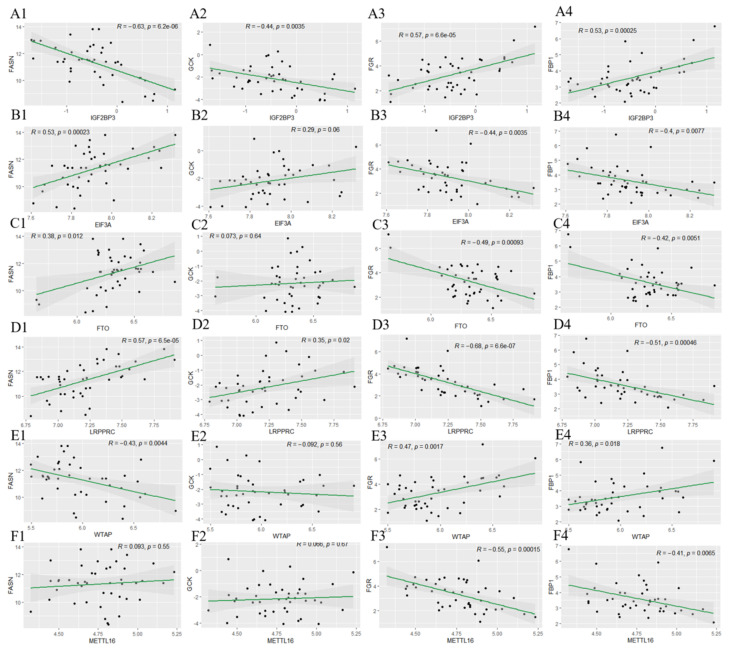
Correlations between *FGR*, *FBP1*, *FASN*, and *GCK* expression and m^6^A modification. The relationships of *FGR*, *FBP1*, *FASN*, and *GCK* expression with *IGF2BP3* (**A1**–**A4**), *EIF3A* (**B1**–**B4**), *FTO* (**C1**–**C4**), *LRPPRC* (**D1**–**D4**), *WTAP* (**E1**–**E4**), and *METTL14* (**F1**–**F4**) expression are shown.

m^6^A modification plays a vital role in the progression of T2D. To explore its involvement in IR, the GSE174475 dataset was used to investigate the relationship between the expression of *FASN*, *GCK*, *FBP1*, and *FGR* and that of 15 m^6^A-related genes in IR [27]. The findings revealed that the expression of these four key genes was significantly correlated with that of *IGF2BP3*, *FTO*, *EIF3A*, *WTAP*, *METTL16*, and *LRPPRC* (*p* < 0.05; Figure 10). Specifically, *FASN* expression was significantly positively correlated with *FTO*, *EIF3A*, and *LRPPRC* expression *(p <* 0.05) and negatively correlated with *IGF2BP3* and *WTAP* expression (*p <* 0.05). The expression of *GCK* was significantly positively correlated with that of *EIF3A* and *LRPPRC* (*p <* 0.05) and negatively correlated with that of *WTAP* (*p <* 0.05). *FGR* expression was significantly positively correlated with *IGF2BP3* and *WTAP* expression (*p <* 0.05) and negatively correlated with *FTO*, *EIF3A*, *METTL16*, and *LRPPRC* expression (*p <* 0.05). The expression of *FBP1* was significantly positively correlated with that of *IGF2BP3* and *WTAP* (*p <* 0.05) and negatively correlated with that of *FTO*, *EIF3A*, *METTL16*, and *LRPPRC* (*p <* 0.05). These results suggest that the changes in expression of the *candidate* genes may be associated with m^6^A modification.

Based on the results on the ability of the *FASN* to predict IR as a biomarker and the correlation with m^6^A modification, *m*eRIP-qPCR was conducted to assess *FASN* methylation levels in clinical samples. The results showed that the methylation levels of *FASN* in adipose tissue of patients with IR were decreased (Figure 9B, *p* < 0.05). Additionally, we found *FASN* mRNA expression was positively correlated with the methylation levels of *FASN* (r = 0.359, *p* = 0.001) in all participants (Figure 9C). These data suggested that the downregulation of FASN may be caused by the decrease in the methylation of FASN in IR.

## 4. Discussion

To the best of our knowledge, this is the first study to identify metabolism-related hub genes associated with IR and the role of m^6^A modification in the pathogenesis of this condition. Additionally, we attempted to validate these essential genes in clinical adipose tissue samples. The findings offer a novel perspective for understanding the molecular basis of IR in adipocytes.

The adipose tissue transcriptome was selected because adipocytes are essential regulators of metabolic homeostasis and play an indispensable regulatory role in IR. Our study had a relatively large transcriptome sample size, being based on the GSE174475 RNA-seq dataset, which includes tissue samples from 27 patients with IR and 16 healthy individuals. Compared with other datasets, GSE174475 comprises transcriptomes in adipocytes rather than in whole adipose tissue, thus eliminating the effect of many non-adipocyte cells in adipose tissue.

Of the 344 DEGs identified in the GSE174475 dataset, 69 were identified in the GeneCards database to encode metabolism-related proteins. GO functional enrichment analysis revealed that these MP-DEGs were enriched in the BP categories regulation of hormone levels and hormone metabolic process, the CC category low-density lipoprotein particle, and the MF categories carboxylic acid transmembrane transporter activity and organic acid transmembrane transporter activity. *SLC27A2*, *FASN*, *ELOVL6*, *SLC16A9*, *SLC6A12*, and *SDS* were all enriched in various biological processes according to the GO enrichment circle map. These genes might be more significant than others in IR. KEGG enrichment analysis showed that the downregulated MP-DEGs were primarily enriched in the insulin signaling, IR, and AMPK signaling pathways. Studies have shown that AMPK plays a major role in regulating cellular energy balance [28]. Therefore, the results indicate that the development of IR and T2D may be regulated by these metabolism-related proteins through their interference with the insulin signaling pathway or energy balance.

Using the PPI network of MP-DEGs and the top 10 hub genes assessed by 10 topological approaches in CytoHubba, we discovered that *FASN*, *GCK*, *FGR*, *FBP1*, *GYS2*, *PNPLA3*, *MOGAT1*, *SLC27A2*, *PNPLA3*, and *ELOVL6* were all simultaneously included in the functional gene modules created by MCODE. These 10 hub genes were highly correlated with IR. The logistic LASSO model can be used to select a greater and more accountable set of predictors from the regression’s massive and underlying multicollinearity set of variables [25]. Through LASSO regression analysis, the 10 hub genes were reduced to three key genes, namely, *GCK*, *FBP1*, and *FGR*. *FASN* was found in the functional module, being a downregulated gene with the highest MCC score (the most accurate method). These four genes were included in the ROC curve analysis, whereupon all four of them showed reasonable diagnostic value for IR, indicating that they are potential biomarkers for the early detection and prevention of T2D. Consistent with our study results, *FBP1* has been previously suggested as a biomarker for the early detection of T2D [29]. *GCK* encodes glucokinase, which catalyzes the phosphorylation of glucose to glucose-6-phosphate. This enzyme plays an indispensable role in the regulation of glucose metabolism and insulin secretion in pancreatic β-cells [30]. Additionally, it was noted in a review that polymorphisms in *GCK* are risk predictors of T2D [31]. A study on building specific biosignatures via machine learning showed that *GCK* methylation was the major feature in two study models and could serve as a biomarker of T2D [32]. Our present study showed that low *GCK* expression in IR was related to early diagnosis.

In our validation of the clinical samples, we found the lower expression of *FASN* has a certain diagnostic value for IR. Many studies have indicated that *FASN* can be used as a biomarker for the diagnosis and prognosis of various cancers, such as triple-negative breast cancer, gastric cancer, and glandular cancer [33,34,35,36,37,38]. Decreased *FASN* expression in the subcutaneous adipose tissue of obese individuals and mice with IR or T2D has been reported [39,40,41]. Similarly, a study suggests that *FASN* can be used as a biomarker for IR, and extracellular FASN has the potential to become an alternative biomarker for the condition [42]. Moreover, studies have shown a correlation of *FASN* mRNA expression with its protein levels in visceral and subcutaneous adipose tissues as well as with impaired insulin sensitivity [40]. The expression and activity of FASN are influenced by insulin [42,43,44], and our results also showed a negative correlation between *FASN* and insulin. Therefore, we speculate that insulin decreases *FASN* mRNA expression by regulating transcription. *FASN* encodes fatty acid synthase, which plays an important role in lipid metabolism as the primary enzyme involved in the de novo synthesis of fatty acids [43,45]. A study by Sun et al. revealed that FTO regulates hepatic lipogenesis via FTO-dependent m^6^A demethylation in FASN mRNA [46]. Additionally, it was recently demonstrated that METTL3 inhibits hepatic insulin sensitivity via the N6-methylation of *FASN* mRNA and promotes fatty acid metabolism [18]. N6-methyladenosine (m^6^A) is a methylation modification formed by the catalysis of a methyltransferase at the sixth nitrogen atom, which regulates gene expression by modulating RNA degradation, splicing, export, and translation [47]. The m^6^A modification is dynamically regulated by an enzyme system, which mainly includes three types of proteins: writers that catalyze m^6^A methylation, erasers that catalyze demethylation, and readers that recognize methylation [48]. Changes in the m^6^A level on a specific gene do not always correlate with changes in the total m^6^A level. This is because the m^6^A modification on a specific gene is dynamically regulated by different methyltransferases (“writers”), demethylases (“erasers”), and binding proteins (“readers”), and each gene has specificity [49]. Our study revealed that *FASN* expression was downregulated in IR and that *FASN* mRNA expression was positively correlated with the methylation levels of *FASN*. The downregulation of *FASN* may be caused by the decrease in the methylation of *FASN* in IR. These results suggest that the m^6^A modification of *FASN* may contribute to the development of IR and T2D. The genes of *FASN* that serve as potential biomarkers of IR should be studied in a larger population, and the role of m^6^A modification in the pathogenesis should be validated in vivo and in vitro.

The current study had several limitations. First, the clinical samples were designed for a case-control study. Second, the validation sample was from a single-center case-control study. Cohort and nested case-control studies with more samples are required to verify the predictive value of these biomarkers. And body composition data such as body fat and muscle mass should also be analyzed in future studies. Moreover, in vivo and in vitro molecular mechanism studies should be carried out to validate how *FASN* is regulated by m^6^A modification in IR.

## 5. Conclusions

In this study, 69 MP-DEGs were screened from the gene expression profiles of human adipose tissues with IR. Four key MP-DEGs were found to distinguish the IR condition. Moreover, the expression of *FASN* and *GCK* in clinical samples was validated. Additionally, we found the downregulation of *FASN* may be caused by the decrease in the methylation of *FASN* in IR. These results will help toward the identification of reliable biomarkers for the early detection and prevention of T2D.

## Figures and Tables

**Table 1:** Correlation of *FASN* and *GCK m*RNA expression and clinical parameters.

	FASN	GCK
FPG, mmol/L	−0.309 **	−0.230 *
HbA1c, %	−0.296 **	−0.096
TC, mmol/L	0.059	−0.011
TG, mmol/L	0.153	0.033
HDL, mmol/L	−0.121	0.060
LDL, mmol/L	0.081	−0.080
Homa-IR	−0.279 **	−0.220 *
BMI	0.097	0.144
FIns, mIU/L	−0.325 **	−0.191

“*” *P* < 0.05; “**” *P* < 0.01.

**Table 2:** The diagnostic capability of *FASN* and *GCK m*RNA for IR.

	AUC (95% CI)	Sensitivity (%)	Specificity (%)	*p*
FASN	0.78 (0.69–0.80)	85.7	65.4	0.000
GCK	0.63 (0.52–0.74)	88.1	40.4	0.028

## Data Availability

The data presented in this study are not publicly available due to confidentiality reasons. The datasets are available from the corresponding author upon reasonable request.

## Supplementary Materials

The following supporting information can be downloaded at: https://www.mdpi.com/article/10.3390/nu15081839/s1, File S1: Design of the study of 343 differentially expressed genes (DEGs) between the insulin resistance (IR) and control groups in the GSE174475 dataset; File S2: 69 differentially expressed genes of metabolism-related proteins (MP-DEGs); File S3: Gene ontology (GO) and Kyoto Encyclopedia of Genes and Genomes (KEGG) enrichment analyses of the 69 differentially expressed genes of metabolism-related proteins (MP-DEGs); File S4: Top 10 differentially expressed genes of metabolism-related proteins (MP-DEGs) identified using 10 topological analysis methods of CytoHubba; Table S1: The Primer sequences used in the study; Table S2: Basic information of samples for GSE174475 used in the study; Table S3: Clinical information of samples used in the study; Table S4: Logistic regression models analysis of insulin resistance; Figure S1:. ROC curves for FASN and GCK.

## References

[1] Tabák A.G., Herder C., Rathmann W., Brunner E.J., Kivimäki M. (2012). Prediabetes: A high-risk state for diabetes development. Lancet.

[2] Knowler W.C., Fowler S.E., Hamman R.F., Christophi C.A., Hoffman H.J., Brenneman A.T., Brown-Friday J.O., Goldberg R., Venditti E., Nathan D.M. (2009). 10-year follow-up of diabetes incidence and weight loss in the Diabetes Prevention Program Outcomes Study. Lancet.

[3] Petersen M.C., Shulman G.I. (2018). Mechanisms of Insulin Action and Insulin Resistance. Physiol. Rev..

[4] Staten M.A., Stern M.P., Miller W.G., Steffes M.W., Campbell S.E. (2010). Insulin assay standardization: Leading to measures of insulin sensitivity and secretion for practical clinical care. Diabetes Care.

[5] Dranse H.J., Waise T.M.Z., Hamr S.C., Bauer P.V., Abraham M.A., Rasmussen B.A., Lam T.K.T. (2018). Physiological and therapeutic regulation of glucose homeostasis by upper small intestinal PepT1-mediated protein sensing. Nat. Commun..

[6] Gustafson B., Hedjazifar S., Gogg S., Hammarstedt A., Smith U. (2015). Insulin resistance and impaired adipogenesis. Trends Endocrinol. Metab..

[7] Burhans M.S., Hagman D.K., Kuzma J.N., Schmidt K.A., Kratz M. (2018). Contribution of Adipose Tissue Inflammation to the Development of Type 2 Diabetes Mellitus. Compr. Physiol..

[8] Roden M., Shulman G.I. (2019). The integrative biology of type 2 diabetes. Nature.

[9] Hu E., Liang P., Spiegelman B.M. (1996). AdipoQ is a novel adipose-specific gene dysregulated in obesity. J. Biol. Chem..

[10] Dua A., Hennes M.I., Hoffmann R.G., Maas D.L., Krakower G.R., Sonnenberg G.E., Kissebah A.H. (1996). Leptin: A significant indicator of total body fat but not of visceral fat and insulin insensitivity in African-American women. Diabetes.

[11] Randle P.J., Garland P.B., Hales C.N., Newsholme E.A. (1963). The glucose fatty-acid cycle. Its role in insulin sensitivity and the metabolic disturbances of diabetes mellitus. Lancet.

[12] Hrdlickova R., Toloue M., Tian B. (2017). RNA-Seq methods for transcriptome analysis. Wiley Interdiscip. Rev. RNA.

[13] Kleinstein S.E., McCorrison J., Ahmed A., Hasturk H., Van Dyke T.E., Freire M. (2021). Transcriptomics of type 2 diabetic and healthy human neutrophils. BMC Immunol..

[14] Herder C., Karakas M., Koenig W. (2011). Biomarkers for the prediction of type 2 diabetes and cardiovascular disease. Clin. Pharmacol. Ther..

[15] Padilla-Martinez F., Wojciechowska G., Szczerbinski L., Kretowski A. (2021). Circulating Nucleic Acid-Based Biomarkers of Type 2 Diabetes. Int. J. Mol. Sci..

[16] Yang Y., Shen F., Huang W., Qin S., Huang J.T., Sergi C., Yuan B.F., Liu S.M. (2019). Glucose Is Involved in the Dynamic Regulation of m6A in Patients with Type 2 Diabetes. J. Clin. Endocrinol. Metab..

[17] De Jesus D.F., Zhang Z., Kahraman S., Brown N.K., Chen M., Hu J., Gupta M.K., He C., Kulkarni R.N. (2019). m(6)A mRNA Methylation Regulates Human β-Cell Biology in Physiological States and in Type 2 Diabetes. Nat. Metab..

[18] Xie W., Ma L.L., Xu Y.Q., Wang B.H., Li S.M. (2019). METTL3 inhibits hepatic insulin sensitivity via N6-methyladenosine modification of Fasn mRNA and promoting fatty acid metabolism. Biochem. Biophys. Res. Commun..

[19] Emont M.P., Jacobs C., Essene A.L., Pant D., Tenen D., Colleluori G., Di Vincenzo A., Jørgensen A.M., Dashti H., Stefek A. (2022). A single-cell atlas of human and mouse white adipose tissue. Nature.

[20] Ritchie M.E., Phipson B., Wu D., Hu Y., Law C.W., Shi W., Smyth G.K. (2015). limma powers differential expression analyses for RNA-sequencing and microarray studies. Nucleic Acids Res..

[21] Uhlén M., Fagerberg L., Hallström B.M., Lindskog C., Oksvold P., Mardinoglu A., Sivertsson Å., Kampf C., Sjöstedt E., Asplund A. (2015). Proteomics. Tissue-based map of the human proteome. Science.

[22] Szklarczyk D., Gable A.L., Lyon D., Junge A., Wyder S., Huerta-Cepas J., Simonovic M., Doncheva N.T., Morris J.H., Bork P. (2019). STRING v11: Protein-protein association networks with increased coverage, supporting functional discovery in genome-wide experimental datasets. Nucleic Acids Res..

[23] Bader G.D., Hogue C.W. (2003). An automated method for finding molecular complexes in large protein interaction networks. BMC Bioinform..

[24] Chin C.H., Chen S.H., Wu H.H., Ho C.W., Ko M.T., Lin C.Y. (2014). cytoHubba: Identifying hub objects and sub-networks from complex interactome. BMC Syst. Biol..

[25] McEligot A.J., Poynor V., Sharma R., Panangadan A. (2020). Logistic LASSO Regression for Dietary Intakes and Breast Cancer. Nutrients.

[26] Polymeris A., Papapetrou P.D. (2022). Anthropometric indicators of insulin resistance. Hormones.

[27] Li Y., Xiao J., Bai J., Tian Y., Qu Y., Chen X., Wang Q., Li X., Zhang Y., Xu J. (2019). Molecular characterization and clinical relevance of m(6)A regulators across 33 cancer types. Mol. Cancer.

[28] Carling D. (2017). AMPK signalling in health and disease. Curr. Opin. Cell Biol..

[29] Wang N., Zhang S., Zhu F., Yang Y., Chen L., Lü P., Yu L., Chen K. (2019). Proteomic Study on the New Potential Mechanism and Biomarkers of Diabetes. Proteom. Clin. Appl..

[30] Osbak K.K., Colclough K., Saint-Martin C., Beer N.L., Bellanné-Chantelot C., Ellard S., Gloyn A.L. (2009). Update on mutations in glucokinase (GCK), which cause maturity-onset diabetes of the young, permanent neonatal diabetes, and hyperinsulinemic hypoglycemia. Hum. Mutat..

[31] Li C., Yang Y., Liu X., Li Z., Liu H., Tan Q. (2020). Glucose metabolism-related gene polymorphisms as the risk predictors of type 2 diabetes. Diabetol. Metab. Syndr..

[32] Karaglani M., Panagopoulou M., Cheimonidi C., Tsamardinos I., Maltezos E., Papanas N., Papazoglou D., Mastorakos G., Chatzaki E. (2022). Liquid Biopsy in Type 2 Diabetes Mellitus Management: Building Specific Biosignatures via Machine Learning. J. Clin. Med..

[33] Cruz M.D., Wali R.K., Bianchi L.K., Radosevich A.J., Crawford S.E., Jepeal L., Goldberg M.J., Weinstein J., Momi N., Roy P. (2014). Colonic mucosal fatty acid synthase as an early biomarker for colorectal neoplasia: Modulation by obesity and gender. Cancer Epidemiol. Biomark. Prev..

[34] Fazli H.R., Moradzadeh M., Mehrbakhsh Z., Sharafkhah M., Masoudi S., Pourshams A., Mohamadkhani A. (2021). Diagnostic Significance of Serum Fatty Acid Synthase in Patients with Pancreatic Cancer. Middle East J. Dig. Dis..

[35] Jiang W., Xing X.L., Zhang C., Yi L., Xu W., Ou J., Zhu N. (2021). MET and FASN as Prognostic Biomarkers of Triple Negative Breast Cancer: A Systematic Evidence Landscape of Clinical Study. Front. Oncol..

[36] Rabionet M., Polonio-Alcalá E., Relat J., Yeste M., Sims-Mourtada J., Kloxin A.M., Planas M., Feliu L., Ciurana J., Puig T. (2021). Fatty acid synthase as a feasible biomarker for triple negative breast cancer stem cell subpopulation cultured on electrospun scaffolds. Mater. Today Biol..

[37] Rhode P., Mehdorn M., Lyros O., Kahlert C., Kurth T., Venus T., Schierle K., Estrela-Lopis I., Jansen-Winkeln B., Lordick F. (2021). Characterization of Total RNA, CD44, FASN, and PTEN mRNAs from Extracellular Vesicles as Biomarkers in Gastric Cancer Patients. Cancers.

[38] Ricklefs F.L., Maire C.L., Matschke J., Dührsen L., Sauvigny T., Holz M., Kolbe K., Peine S., Herold-Mende C., Carter B. (2020). FASN Is a Biomarker Enriched in Malignant Glioma-Derived Extracellular Vesicles. Int. J. Mol. Sci..

[39] Nadler S.T., Stoehr J.P., Schueler K.L., Tanimoto G., Yandell B.S., Attie A.D. (2000). The expression of adipogenic genes is decreased in obesity and diabetes mellitus. Proc. Natl. Acad. Sci. USA.

[40] Menendez J.A., Vazquez-Martin A., Ortega F.J., Fernandez-Real J.M. (2009). Fatty Acid Synthase: Association with Insulin Resistance, Type 2 Diabetes, and Cancer. Clin. Chem..

[41] Sievert H., Krause C., Geißler C., Grohs M., El-Gammal A.T., Wolter S., Mann O., Lehnert H., Kirchner H. (2020). Epigenetic Downregulation of FASN in Visceral Adipose Tissue of Insulin Resistant Subjects. Exp. Clin. Endocrinol. Diabetes.

[42] Fernandez-Real J.M., Menendez J.A., Moreno-Navarrete J.M., Blüher M., Vazquez-Martin A., Vázquez M.J., Ortega F., Diéguez C., Frühbeck G., Ricart W. (2010). Extracellular fatty acid synthase: A possible surrogate biomarker of insulin resistance. Diabetes.

[43] Hillgartner F.B., Salati L.M., Goodridge A.G. (1995). Physiological and molecular mechanisms involved in nutritional regulation of fatty acid synthesis. Physiol. Rev..

[44] Moreno-Indias I., Tinahones F.J. (2015). Impaired adipose tissue expandability and lipogenic capacities as ones of the main causes of metabolic disorders. J. Diabetes Res..

[45] Zhang J., Song Y., Shi Q., Fu L. (2021). Research progress on FASN and MGLL in the regulation of abnormal lipid metabolism and the relationship between tumor invasion and metastasis. Front. Med..

[46] Sun D., Zhao T., Zhang Q., Wu M., Zhang Z. (2021). Fat mass and obesity-associated protein regulates lipogenesis via m(6) A modification in fatty acid synthase mRNA. Cell Biol. Int..

[47] Zhou J., Wan J., Gao X., Zhang X., Jaffrey S.R., Qian S.B. (2015). Dynamic m(6)A mRNA methylation directs translational control of heat shock response. Nature.

[48] Wang X., Lu Z., Gomez A., Hon G.C., Yue Y., Han D., Fu Y., Parisien M., Dai Q., Jia G. (2014). N6-methyladenosine-dependent regulation of messenger RNA stability. Nature.

[49] Zhao T., Sun D., Xiong W., Man J., Zhang Q., Zhao M., Zhang Z. (2023). N(6)-methyladenosine plays a dual role in arsenic carcinogenesis by temporal-specific control of core target AKT1. J. Hazard. Mater..

