# Supplementary material for: Identification of Metabolism-Related Proteins as Biomarkers of Insulin Resistance and Potential Mechanisms of m6A Modification
Source: Nutrients. 2023 Apr 11;15(8):1839. doi: 10.3390/nu15081839 (PMC10146912; doi:10.3390/nu15081839)
Supplement: Supplementary file 1 [file nutrients-15-01839-s001.zip › supplementary material/Supplementary material.docx]

**Supplementary material**

**Supplement Excell**

**Supplement Excell 1. Design of the study of 343 differentially expressed genes (DEGs) between the insulin resistance (IR) and control groups in the GSE174475 dataset.**

**Supplement Excell 2. 69 differentially expressed genes of metabolism-related proteins (MP-DEGs).**

**Supplement Excell 3. Gene ontology (GO) and Kyoto Encyclopedia of Genes and Genomes (KEGG) enrichment analyses of the 69 differentially expressed genes of metabolism-related proteins (MP-DEGs).**

**Supplement Excell 4. Top 10 differentially expressed genes of metabolism-related proteins (MP-DEGs) identified using 10 topological analysis methods of CytoHubba.**

**Supplement Table**

**Supplement Table 1. The Primer sequences used in the study**

**Supplement Table 2. Basic information of samples for GSE174475 used in the study**

**Supplement Table 3. Clinical information of samples used in the study**

**Supplement Table 4. Logistic regression models analysis of insulin resistance**

**Supplement Figure**

**Supplement Figure 1. ROC curves for *FASN* and *GCK***

Supplement table 1. The primer sequences

| RNA | Primer sequences | ID |
| --- | --- | --- |
| FASN | F: 5'AAGGACCTGTCTAGGTTTGATGC3'  R: 5' TGGCTTCATAGGT‐GACTTCCA 3' | 2194 |
| GCK | F: 5'GAGGCCGCCAAGAAGGAGAA 3' | 2645 |
|  | R: 5' GTAGTCGAAGAGCATCTCA 3' |  |
| GAPDH | F: 5' GAAGGTCGGAGTCAACGGATT 3' | 2597 |
|  | R: 5' CGCTCCTGGAAGATGGTGAT 3' |  |

Supplement table 2. Basic information of samples for GSE174475 used in the study

| **Characteristic** | **IR (n=27)** | **Control (n=16)** | ***P(t/χ）*** |
| --- | --- | --- | --- |
| Age, yr | 50.63±10.53 | 57.25±12.71 | 0.289 |
| BMI, kg/m² | 30.09±4.93 | 27.17±3.32 | 0.042 |
| Homa-IR, | 5.80±4.79 | 0.70±0.15 | 0.000 |
| TG | 134.11±60.91 | 82.63±43.49 | 0.005 |
| HDL | 54.11±19.12 | 70.50±26.04 | 0.022 |
| LDL | 97.89±35.62 | 93.19±26.92 | 0.651 |
| Gender（%Female） | 27（100%） | 16（100%） | - |

Supplement table 3. Clinical information of samples used in the study

| **Characteristic** | **IR(n=42)** | **Control (n=52)** | ***P(t/χ）*** |
| --- | --- | --- | --- |
| Age, yr | 65.00±5.50 | 63.58.75±8.89 | 0.416 |
| BMI, kg/m² | 25.09±2.66 | 24.41±4.79 | 0.367 |
| HbA1c, % | 6.55±1.09 | 5.41±0.67 | 0.000 |
| FPG, mmol/L | 6.60±1.42 | 4.84±0..80 | 0.000 |
| TC, mmol/L | 3.55±1.21 | 3.32±0.63 | 0.229 |
| TG, mmol/L | 1.33±0.73 | 1.19±0.68 | 0.327 |
| HDL, mmol/L | 0.95±0.23 | 1.01±0.24 | 0.258 |
| LDL, mmol/L | 1.94±0.97 | 1.73±0.47 | 0.161 |
| Homa-IR | 4.27±1.41 | 1.97±039 | 0.000 |
| Gender（%Male） | 39（92.8%） | 43（82.7%） | 0.123 |
| Smoking（%Yes） | 10（23.8%） | 13（25.0%） | 0.89 |
| Drinking（%Yes） | 12（28.6%） | 17（32.7%） | 0.67 |
| Physical activity（% Yes） | 25（59.5%） | 37（71.2%） | 0.24 |
| Salt addiction |  |  | 0.84 |
| Light taste | 16（38.1%） | 23（44.2%） |  |
| Moderate taste | 17（40.5%） | 19（36.5%） |  |
| High-salt taste | 9（21.4%） | 10（19.2%） |  |
| Oil addiction |  |  | 0.46 |
| Light taste | 14（33.3%） | 16（30.8%） |  |
| Moderate taste | 12（28.6%） | 21（40.4%） |  |
| Oily taste | 16（38.1%） | 15（28.8%） |  |
| Sugar addiction |  |  | 0.74 |
| No | 9（21.4%） | 12（23.1%） |  |
| Moderate taste | 12（28.6%） | 18（34.6%） |  |
| High-sugar taste | 21（50.0%） | 22（42.3%） |  |

Abbreviations: HDL-C, high-density lipoprotein cholesterol; LDL-C, low-density lipoprotein cholesterol; TC, total cholesterol; TG, triglyceride.

Supplement table 4. Logistic regression models analysis of insulin resistance

|  | OR | (95%CI) | *P*值 |
| --- | --- | --- | --- |
| *FASN* |  |  |  |
| Model 1 | 0.154 | (0.051-0.464) | 0.001 |
| Model 2 | 0.168 | (0.054-0.519) | 0.002 |
| Model 3 | 0.152 | (0.047-0.488) | 0.002 |
| Model 4 | 0.121 | (0.034-0.432) | 0.001 |
| *GCK* |  |  |  |
| Model 1 | 0.265 | (0.092-0.763) | 0.014 |
| Model 2 | 0.280 | (0.096-0.819) | 0.020 |
| Model 3 | 0.254 | (0.085-0.761) | 0.014 |
| Model 4 | 0.258 | (0.083-0.806) | 0.020 |

Model 1：Univariate regression model, Model 2：：adjusting for age and gender, Model 3: adjusting for age, gender and BMI, Model 4: adjusting for age, gender, BMI, smoking, drinking, physical activity, salt addiction, oil addiction and sugar addiction.


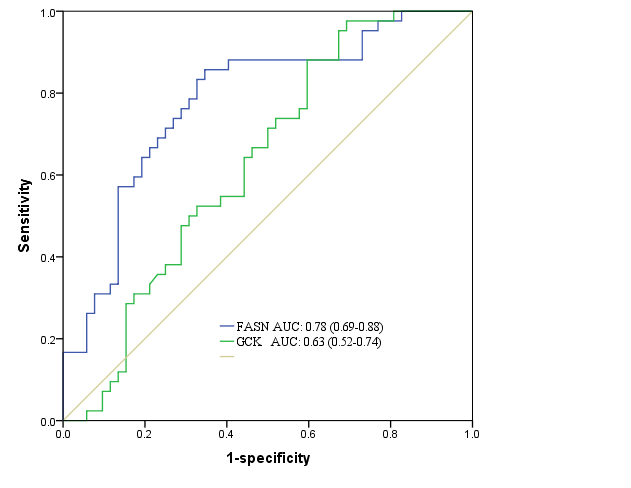


Supplement Fig. ROC curves for *FASN* and *GCK* (AUC: 0.78 and 0.63, respectively); *P<*0.05*.*
